# Application of DSM–BIA in dry weight assessment in continuous ambulatory peritoneal dialysis

**DOI:** 10.1007/s11255-022-03281-7

**Published:** 2022-07-04

**Authors:** Qi Chen, Zheng Wang, Na Liu, Shujuan Mu, Peng Guo, Shichao Li, Jingwei Zhou, Yan Li

**Affiliations:** 1 Department of Nephrology, Dongzhimen Hospital, Beijing University of Chinese Medicine, No. 5 Haiyuncang, Dongcheng District, Beijing, 100000 China; 2 Ningxia Hospital of Traditional Chinese Medicine and Chinese Medicine Research Institute, Ningxia, China; 3 Department of Nephrology, The South District of Guanganmen Hospital, Chinese Academy of Chinese Medical Science, Beijing, China

**Keywords:** Dry weight assessment, Direct segmental multi-frequency BIA, Continuous ambulatory peritoneal dialysis, Fluid management

## Abstract

**Objectives:**

Dry weight assessment (DWA) is an important part of dialysis and fluid management in patients receiving renal replacement therapy. With the development of bioimpedance analysis (BIA), the development of the direct segmental multi-frequency BIA (DSM–BIA) has provided a more convenient measure for DWA of dialysis patients, but its accuracy remains unclear. This study was designed to evaluate the application of DSM–BIA in DWA of continuous ambulatory peritoneal dialysis (CAPD) patients.

**Design:**

This is a cross-sectional study. Using the conventional BIA as a reference, we examined the accuracy of the DSM–BIA technique for assessing dry weight in CAPD patients and analyzed the potential factors influencing their fluid volume status.

**Setting and participants:**

A total of 31 patients with end-stage renal disease receiving CAPD and 310 healthy volunteers were recruited for this study.

**Methods:**

The intraclass correlation coefficients (ICC) and Bland–Altman plots were used to assess the consistency between DSM–BIA and the conventional BIA for DWA. Univariate and multivariate linear regression analyses were used to explore the influencing factors associated with the edema index.

**Results:**

DSM–BIA and the conventional BIA technology were consistent in DWA in CAPD patients (ICC female 0.972, ICC male 0.882, ICC total 0.960). Similarly, Bland–Altman plots showed good agreements between the two methods in DWA for both genders. Univariate and multivariate linear regression analysis showed both eGFR level (*P* = 0.04) and serum NT-pro BNP concentration (*P* = 0.007) were positively correlated with the ratio of extracellular water to total body water (ECW/TCW).

**Conclusions:**

DSM–BIA in DWA has good accuracy in clinical applications and has potential application value for fluid volume management in CAPD patients.

**Supplementary Information:**

The online version contains supplementary material available at 10.1007/s11255-022-03281-7.

## Introduction

Adequate control of extracellular fluid volume in patients receiving renal replacement therapy (RRT) is one of the main objectives of dialysis therapy, so clinicians need to make an accurate assessment of the patient's fluid status. Dry weight assessment (DWA), defined as a patient's post-dialysis weight while maintaining normal blood pressure during dialysis without the use of hypertension medications [1], is an important method for fluid volume management in dialysis patients. Inaccurate DWA will lead to corresponding hypervolemia (headache, hypertension, dyspnea, and orthopnea) or hypovolemia (cramps, fatigue, and orthostatic hypotension). However, low sensitivity to these symptoms and high variability among patients tend to lead to more severe volume depletion or volume overload, which are associated with a higher risk of cardiovascular events and death [2].

Traditionally, DWA has been achieved through some clinical examinations, including the usual blood pressure measurement and target dry weight titration until normal blood volume, which is entirely dependent on the expertise and skills of the clinician [3]. The clinical assessment is far more complex and includes vascular stiffness, cardiac insufficiency, hypoproteinemia, and morbidity, as well as the desire to better preservation of residual kidney function, so more objective tools are needed to assess dry weight for patients receiving RRT.

Several objective methods have been proposed to support the correct estimation of dry weight in dialysis patients, including inferior vena cava ultrasound, radionuclide dilution techniques, and echocardiography. However, these methods have many disadvantages, such as time consumption, difficulty in daily practice, and inability to quantify excess or deficiency of body fluids. So far, most dialysis centers still rely on subjective clinical criteria to assess dry weight. Studies have concluded that bioelectrical impedance analysis (BIA) and bioimpedance spectroscopy (BIS) may be useful for the clinical assessment of fluid overload [3, 4], in patients receiving RRT due to their objectivity and portability. Both BIA and BIS are mainly based on the electrical conductivity of body water composition, calculating the water volume of the body by measuring the impedance value of water by turning on a small amount of alternating current. The difference between BIA and BIS is that BIA is mainly based on built-in regression equations, while BIS is based on Cole modeling and mixture theories [5]. The defect of the early conventional BIA is that the human body is regarded as a simple cylinder and only two electrodes are used to measure the electrical impedance from the wrist to the ankle of the unilateral limb, ignoring the physiological complexity of the human body structure [6]. Although the traditional single-frequency BIA method is not considered to be superior to BIS, with the improvement and development of direct segmental multi-frequency BIA (DSM–BIA) technology, DSM–BIA has certain advantages in the evaluation of human body composition. DSM–BIA directly measures the trunk impedance without using the empirical estimation, which ensures the high repeatability and accuracy of the results [7]. Recently, DSM–BIA has shown superior results in the estimation of body composition [8] and fluid volume management of hemodialysis patients [9], but the results in DWA of continuous ambulatory peritoneal dialysis (CAPD) patients are not very clear. Therefore, we conducted this—study to investigate the accuracy of DSM–BIA in DWA of continuous ambulatory peritoneal dialysis (CAPD) patients and to explore the related factors affecting fluid status in patients with CAPD.

## Methods

### Study design and population

The study design was a cross-sectional study of patients with ESRD who had received CAPD for more than 3 months. Participants of CAPD were mainly from patients regularly followed up in the dialysis center of our hospital. Inclusion criteria were as follows: (1) the patient received CAPD due to renal function decline to stage 5 of chronic kidney disease, and the duration of CAPD was longer than 3 months; (2) all patients were older than 18 years and younger than 75 years; (3) serum albumin ≥ 30 g/L; and (4) body mass index (BMI) ≤ 40 kg/m^2^. Exclusion criteria were as follows: (1) Patients with heart failure with cardiac function ≥ NYHA III grade, and patients with liver failure or advanced tumor; (2) patients with skin infection of limbs; (3) patients with metal prosthesis or pacemaker installed in their bodies; (4) vascular diseases of lower extremities (such as arteriosclerosis obliterans, diabetic foot and varicose veins); and (5) a history of central venous and inferior vena cava stenosis. The research program was approved by the Institutional Review Board. Each patient signed a written informed consent to participate in the study before being assessed.

### Measurement of dry weight assessment by body composition analyzer

Measurement of dry weight assessment was performed using the Inbody S10 Multifrequency Bioelectrical Impedance analyzer (Biospace, Seoul, Korea). All participants were required to empty the gastrointestinal tract, avoid strenuous exercise or showers, and stop the infusion of nutrient solution for more than 3 h before measurement. The indoor temperature should be kept at 20–25 ℃. All measurements were taken under the guidance of trained research nurses.

### Direct segmental multi-frequency bioelectrical impedance analysis (DSM–BIA)

The DSM–BIA technique assumes that the human body consists of five interconnected cylinders that measure the impedance of the subject's trunk, arms, and legs at six different frequencies (1, 5, 50, 250, 500, and 1000 kHz). The electrical frequency spectrum was used to predict the intracellular water (ICW) and extracellular water (ECW) of total body water (TBW) in different body parts, and immediate and extensive quantitative values of various body composition parameters were also measured.

### Conventional bioelectric impedance analysis

Conventional bioelectric impedance analysis relies on formulas to estimate dry weight. In this study, we used the formula proposed by Chamney to directly calculate dry weight using the results of InbodyS10 Multifrequency Bioelectrical Impedance analyzer [10], which is based on the assumption that patients will gradually approach the volumetric state of a healthy person as the body is dehydrated with dialysis, and the dry weight is calculated when the ECW% in body weight intersects with the slope of the normal value. The Chamney formula is as follows, where ECW% (ECW/TBW%) needs to be obtained from the corresponding normal population in the same BMI area group, Wgt_m_ is the bodyweight of patients before receiving CAPD, and ECW_m_ is the weight of ECW in patients measured by bioelectrical impedance analyzer.

$${\text{Dry weight}}\,\left( {{\text{kg}}} \right)\, = \,({\text{Wgt}}_{{\text{m}}} - {\text{ECW}}_{{\text{m}}} )/({1} - {\text{ECW}}\% )$$

### Echocardiography and laboratory analysis

All patients were given 2.5% peritoneal dialysis solution at night for 8 h. The peritoneal dialysis solution was released after the morning of the second day, and another 2.5% peritoneal dialysis solution was injected. Blood samples were collected 2 h later, and the peritoneal dialysis solution was released 4 h later, and the solution specimens were retained. The blood samples were used to test the blood routine and serum biochemical parameters, and the peritoneal dialysate samples were used to test the corresponding biochemical parameters. Residual estimated glomerular filtration rate (eGFR) was assessed by the estimated slope of renal urea clearance (KRU). Echocardiography was performed in all patients within 3 months after the start of CAPD.

### Statistical analyses

Normally distributed continuous variables are expressed as mean ± standard deviation (SD), skewed continuous variables are expressed as median and interquartile ranges, and categorical variables are expressed as frequency and percentage. The unpaired Student’s *t* test was used to compare the continuous variables with normal distribution, and Mann–Whitney *U* test was used to test the continuous variables with non-normal distribution. The Bland–Altman plots were used to examine the consistency of the two methods in DWA. Pearson correlation was used to quantify the deviations seen on the Bland–Altman curve. To improve the clinical utility, univariate and multivariate linear regression analysis were used to assess the relevant factors influencing patients' fluid volume status. A two-sided *P* < 0.05 was considered statistically significant. GraphPad Prism 8.4.3 (GaphPadSoftware Inc, LaJolla, CA), and SPSS 16.0 (IBM Corp., Armonk, NY) were used for all statistical analyses.

## Results

### Clinical characteristics of study participants

A total of 31 CAPD patients were enrolled in our study, and their clinical characteristics according to gender are summarized in Table 1. As expected, female subjects had significantly lower height, weight, and body surface area than male subjects, but no significant gender differences in body mass index (*P* = 0.07). Moreover, the smoking and drinking histories of females were significantly lower than those of males. It should be noted that in our study, there was a significant imbalance in the solute transport capacity in the peritoneum of males and females, and the detailed distribution of peritoneal transport type is shown in Supplement Table 1.

**Table 1:** Comparisons of clinical characteristics of study participants according to gender

Characteristic	Male (*N* = 21)	Female (*N* = 10)	Total (*N* = 31)	*P* value
Age, years	56.3 ± 10.6	54.9 ± 14.1	55.9 ± 11.9	0.76
Dialysis vintage, months	31.5 ± 18.2	39.2 ± 23.1	34.0 ± 19.6	0.32
History of smoking	14 (67.0%)	1 (10.0%)	15 (48.3%)	0.006
History of drinking	12 (57.1%)	1 (10.0%)	13 (41.9%)	0.02
Height, cm	172.4 ± 3.8	154.5 ± 4.7	166.0 ± 9.4	< 0.001
Weight, kg	71.3 ± 87.6	53.6 ± 6.3	64.8 ± 11.0	< 0.001
BMI, kg/m^2^	24.0 ± 2.6	22.2 ± 2.1	23.3 ± 2.3	0.07
BSA, m^2^	1.8 ± 0.1	1.5 ± 0.1	1.7 ± 0.2	< 0.001
eGFR, ml/min per 1.73 m^2^	5.3 ± 1.7	5.6 ± 1.8	5.4 ± 1.7	0.67
Ccr, ml/min	7.6 ± 2.2	6.5 ± 1.3	5.5 ± 1.8	0.14
PET	0.7 ± 0.1	0.6 ± 0.1	0.6 ± 0.1	0.01
UFV, ml	205.1 ± 85.3	209.1 ± 166.2	215.4 ± 100.1	0.42
Systolic BP, mm Hg	144.6 ± 16.8	142.0 ± 20.7	146.7 ± 17.7	0.72
Diastolic BP, mm Hg	84.5 ± 10.6	84.6 ± 11.0	83.8 ± 10.5	0.98

*BMI* body mass index, *BSA* body surface area, *eGFR* estimated glomerular filtration rate, *Ccr* creatinine clearance rate, *PET* peritoneal equilibration test, *UFV* Ultrafiltration volume, *BP* blood pressure

### Agreements between DSM–BIA and conventional BIA in DWA

We collected data from 310 healthy adults as a reference control group, whose clinical characteristics and body composition analysis results are presented in Supplement Table 2.

The DWA calculated by the DSM–BIA and conventional BIA according to the different genders is shown in Table 2. By comparison, there are good agreements between two methods in DWA for total CAPD patients (intraclass correlation coefficients (ICC) 0.960, 95% CI 0.918–0.981; Table 2) and both genders. Furthermore, it was noted that the agreement of the two dry weight measures in women (ICC 0.970, 95% CI 0.880–0.993; Table 2) was slightly stronger than in men (ICC 0.882, 95% CI 0.709–0.952; Table 2) and in total CAPD patients.

**Table 2:** Dry weight assessment calculated by different methods according to gender

Items	Male (*N* = 21)	Female (*N* = 10)	Total (*N* = 31)
DSM–BIA	70.9 ± 6.2	54.2 ± 5.4	65.5 ± 9.8
Conventional BIA	67.4 ± 7.5	51.9 ± 6.2	62.4 ± 10.1
ICC	0.88 (0.71–0.95)	0.97 (0.88–0.99)	0.96 (0.92–0.98)

*ICC* intraclass correlation coefficient

**Fig. 1:**
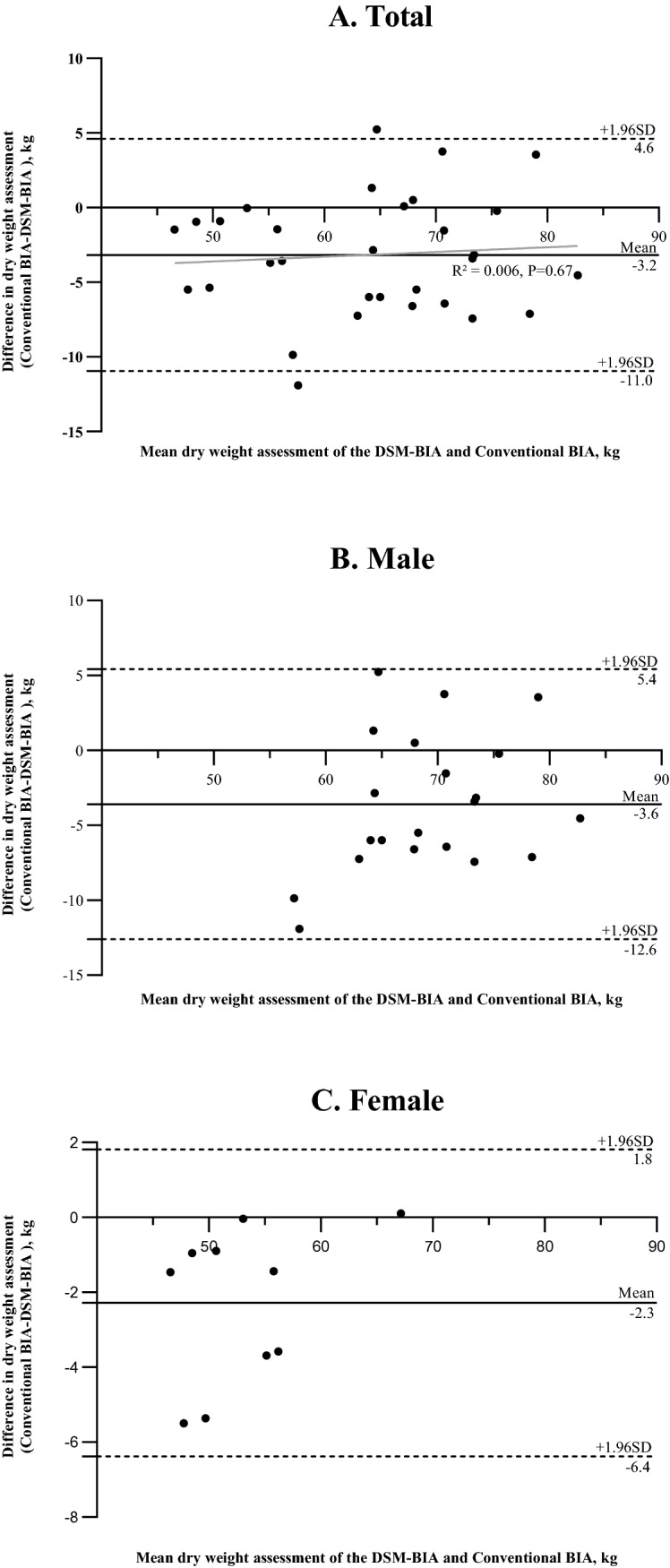
Conventional Bland–Altman plots showing the difference vs. mean value of dry weight assessment on DSM–BIA and conventional BIA for total CAPD patients and both genders. The mean difference and 95% limits of agreement are shown

The Bland–Altman plots were used to visually evaluate the agreement between the DSM–BIA and conventional BIA in DWA, as shown in Fig. 1. For all CAPD patients, 93.5% (29/31) of the scatter plots are within the estimated 95% CI range, Fig. 1A, suggesting good agreement between the two assessment methods in the measurements of DWA. The mean difference of DWA between DSM–BIA and the conventional BIA is 3.2 kg (SD of 4.0 kg), which indicates that DSM–BIA overestimates the dry weight compared to conventional BIA. There is no association was found between the difference (*y*-axis) across the range of – 11.9 to 5.2 kg and the average (*x*-axis) across the range of 46.6–82.7 kg between the two methods for DWA (*R*^2^ = 0.006, *P* = 0.67; Fig. 1A). The Bland–Altman plots were also used to examine the agreement of the two methods in the measurements of DWA in male and female CAPD patients, respectively. The results showed that all the data points (100%) of the two assessment methods are limited in the estimated 95% CI range (Fig. 1B, C), revealing the excellent agreement of the two assessment methods in DWA for both genders.

### Factors associated with edema index in CAPD patients

To determine the possible factors associated with the fluid volume status of patients with CAPD, univariate and multivariate linear regression analyses were performed in the entire CAPD population in the study. The edema index (ECW/TBW) measured by the body composition analyzer was taken as the dependent variable, and the patient's blood test results, dialysate test results, and the parameters of the cardiac function were evaluated by echocardiography were taken as the independent variables. On univariate analysis. as shown in Table 3 and Supplement Table 3, a statistically significant positive linear correlation was found on ECW/TBW with age (*P* = 0.004), estimated glomerular filtration rate (eGFR) (*P* = 0.04), C-reactive protein (*P* = 0.04), NT-pro BNP (*P* = 0.02), left ventricular end-systolic diameter (LVESD) (*P* = 0.03) and left atrial anteroposterior diameter (LAAD) (*P* = 0.005), whereas a statistically significant negative linear correlation was found on ECW/TBW with Platelets (*P* = 0.04), left ventricular ejection fraction (LVEF) (*P* = 0.02), and Left ventricular fractional shortening (LVFS) (*P* = 0.02). No statistically significant correlation was found for other factors in CAPD patients (Supplement Table 3). In multivariable linear stepwise analysis, as can be seen in Table 3, NT-proBNP (*P* = 0.007) and eGFR (*P* = 0.04) were independently and positively correlated with ECW/TCW among the various factors examined. In addition, the *R*^2^ value is 0.409, which means that NT-proBNP and eGFR can explain a 40.9% change of ECW/TCW.

**Table 3:** Linear regression analysis of parameters possibly associated with ECW/TCW

Parameter	ANOVA (F)	Univariate linear regression (Std. *β*)	*P* value	Multivariate linear regression (Std. *β*)	*P* value
Age, year	9.6	136.3	0.004		
Gender	0.4	6.1	0.53		
eGFR, ml/min per 1.73 m^2^	4.5	21.2	0.04	0.003	0.04
BMI, kg/m^2^	0.2	34.4	0.65		
MAP, mmHg	6.9	126.4	0.01		
UFA, ml	0.5	1313.0	0.50		
Dialysis vintage, month	0.1	258.1	0.76		
PET	1.0	1.2	0.32		
Clinical edema	5.6	8.1	0.03		
Blood test results					
Platelets, × 10^9^/L	4.5	– 2070.0	0.04		
C-reactive protein, mg/L	4.5	232.4	0.04		
NT-pro BNP, pg/ml	5.9	16049.0	0.02	< 0.001	0.007
M-mode and two-dimensional echocardiography					
LVESD, mm	5.4	183.9	0.03		
LVEF, %	6.4	– 345.4	0.02		
LVFS, %	7.0	– 201.6	0.01		
LAAD, mm	9.5	199.9	0.005		

*eGFR* estimated glomerular filtration rate, *BMI* body mass index, *MAP* mean arterial pressure, *UFV* Ultrafiltration volume, *PET* peritoneal equilibration test, *LVESD* Left ventricular end systolic diameter, *LVEF* Left ventricular ejection fraction, *LVFS* Left ventricular fractional shortening, *LAAD* Left atrial anteroposterior diameter

## Discussion

This study demonstrated that the DSM–BIA assessment of dry weight is in good agreement with the conventional BIA assessment method in CAPD patients regardless of the gender, and that volume overload control represented as edema index (ECW/TBW ratio) under the guidance of DSM–BIA is closely related to the eGFR level and serum NT-pro BNP concentration in CAPD patients. These results confirmed the value of the application of DSM–BIA in DWA and its usefulness in fluid volume management of CAPD patients.

It is well known that in patients with end-stage renal disease, the increase in systemic sodium and fluid volume is an inevitable consequence of the decrease in renal function, which is followed by the elevation of blood pressure and damage in blood vessels. These factors are closely related to the risk of serious cardiovascular complications [1]. Hypertension control in end-stage renal disease is one of the important objectives of treatment management. Studies have shown that reduced sodium excretion as the decline in renal function leads to hypertension in about 90% of dialysis patients, and that good control of sodium and fluid balance in patients can control hypertension without the use of antihypertensive drugs [11]. Given that positive sodium balance and the resulting hypervolemia are two major causes of hypertension and increased cardiovascular mortality in patients with end-stage renal disease, it is important to accurately assess the amount of fluid discharged from the body during dialysis. Therefore, achieving and maintaining dry weight is an effective strategy to control blood pressure and reduce the risk of cardiovascular events in patients receiving dialysis. Our study confirms that the DSM–BIA method has good accuracy in dry weight assessment in CAPD patients, which will provide clinicians with a more convenient and effective way to assess dry weight in clinical practice. A previous randomized controlled trial (RCT) involving hemodialysis patients showed that the guidance under BIA for dialysis significantly reduced all-cause mortality over 2.5-year follow-up period, and a recent 3-year extended exploratory study of a prospective RCT concluded that DSM–BIA guided fluid management improved hard outcomes in terms of patient survival and non-cardiovascular survival [4]. These findings provide more evidence and confidence for the clinical application of DSM–BIA and merit further study in longer follow-up periods and more carefully designed clinical studies.

BIA is an ideal tool for measuring the impact of interventions aimed at changing fluid status, so it is a good way to routinely monitor fluid status and nutrition in dialysis patients, helping them control fluid overload more effectively and more quickly. Clinicians need to be very cautious and combine clinical judgment in setting target body weight, because there is increasing evidence from bioimpedance data that excessive hydration predicts poorer survival [3, 12]. A cross-sectional study has shown that DSM–BIA provides a good assessment of total body water to obtain an accurate assessment of hydration status [13]. DSM–BIA can accurately measure water in and out of cells and produce an "edema index", the ratio of ECW to TBW. Several cohort studies have shown that ECW/TBW ratio is strongly associated with poorer survival in PD patients, even after adjusting for known confounders, such as comorbidities, inflammation, and hypoalbuminemia [14]. In our study, we observed that a lower ECW/TBW ratio was associated with a lower level of eGFR in CAPD patients. Studies have shown that excessive water itself leads to a decrease in residual renal function [4, 15], so it is necessary to better control fluid overload clinically to better preserve the residual renal function. Importantly, we also found that higher ECW/TBW ratios were strongly associated with higher NT-pro BNP levels. Elevated NT-pro BNP is known to be strongly associated with cardiac abnormalities^16^, such as left ventricular hypertrophy, atrial enlargement, or estimated right ventricular systolic pressure. However, the correlation between cardiac biomarkers and hydration status remained highly heterogeneous, with some studies finding no correlation with hydration status, while others observed a correlation between change in ECW and NT-pro BNP [17], which also was consistent with our results. In addition, we also observed that a lower ECW/TBW ratio was associated with a lower level of eGFR in CAPD patients. Theoretically, overhydration should be associated with a decrease in residual renal function, which seems inconsistent with our findings. However, because this study is a cross-sectional study, there is no causal relationship between ECW/TBW and eGFR, and in CAPD patients, whether the high volume state is related to the decline of residual renal function has been controversial, and fluctuations in volume status are also one of the risk factors for the decline of residual renal function [5], so whether overhydration is related to the decline of residual renal function needs to be further explored by prospective clinical studies. Our findings confirm the consistency of the two DWA methods (the difference is not statistically significant), but this difference of around 3 kg (SD 4 kg) may have some significance for clinical practice. We think that DSM–BIA is a "multi-segment impedance measurement" compared to the " wrist and ankle impedance measurement" used in conventional BIA, which should be able to measure the overall impedance more accurately, but the problem remains that it is probable that the value of these machines consists in tracking hydration status changes rather than assessment of dry weight and the results of the DSM–BIA compared to the clinically assessed DWA still need further research. It is worth noting that although BIA equipment can help evaluate volume overload in CAPD patients, the effect of BIA on short-term hard endpoints of peritoneal dialysis is still not consistent, larger and longer follow-up clinical studies is necessary to confirm the results. Our study also has some other limitations, such as limited number of included patients in this study, the rate imbalance in the solute transport capacity in the peritoneum between baseline sexes, and the lack of assessment of clinical edema be made on the patients. Taken together, our findings suggest that DSM–BIA assessment of dry weight has good accuracy in clinical applications and has potential application value for fluid volume management in CAPD patients. It is necessary to conduct prospective large-scale randomized controlled trials to determine the impact of DSM–BIA guidance on long-term outcomes in CAPD patients.

## Supplementary Information

Below is the link to the electronic supplementary material.

Supplementary file1 (DOCX 173 KB)
