# Supplementary material for: Application of DSM–BIA in dry weight assessment in continuous ambulatory peritoneal dialysis
Source: Int Urol Nephrol. 2022 Jul 4;54(12):3263–70. doi: 10.1007/s11255-022-03281-7 (PMC9605927; doi:10.1007/s11255-022-03281-7)
Supplement: Supplementary file 1 — Supplementary file1 (DOCX 173 KB) [file 11255_2022_3281_MOESM1_ESM.docx]

**Supplement Table 1. Comparisons of clinical Characteristics of Study Participants** **according to gender.**

| **Characteristic** | **Male(N=21)** | **Female(N= 10)** | | **Total (N = 31)** | | **P value** |
| --- | --- | --- | --- | --- | --- | --- |
| **Peritoneal transport type** |  |  | |  | | 0.09 |
| High transport type | 1(3.2%) | 0 | | 1(3.2%) | |  |
| High-average transport type | 9(29.0%) | 1(3.2%) | | 10(32.2%) | |  |
| Average transport type | 1(3.2%) | 0 | | 1(3.2%) | |  |
| Low-average transport type | 10(32.2%) | 7(22.5%) | | 17(54.8%) | |  |
| Low transport type | 0 | 2(6.4%) | | 2(6.4%) | |  |
| **Blood test results** |  |  | |  | |  |
| White blood cells, ×10^9^/L | 6.8±2.2 | 6.5±1.4 | | 6.7±2.0 | | 0.75 |
| Red blood cells, ×10^12^/L | 3.6±0.6 | 3.5±0.7 | | 3.6±0.6 | | 0.57 |
| Hemoglobin, g/L | 109.6±26.3 | 112.4±24.6 | | 110.5±25.8 | | 0.79 |
| Platelets, ×10^9^/L | 206.4±62.1 | 221.4±106.5 | | 211.2±79.5 | | 0.64 |
| C-reactive protein, mg/L | 5.3±6.1 | 6.4±9.4 | | 5.6±7.3 | | 0.75 |
| Kalium, mmol/L | 4.2±0.6 | 3.9±0.6 | | 4.1±0.6 | | 0.31 |
| Natrium, mmol/L | 139.5±2.2 | 139.9±3.0 | | 139.6±2.4 | | 0.68 |
| Calcium, mmol/L | 2.3±0.2 | 2.5±0.3 | | 2.4±0.2 | | 0.13 |
| Phosphorus, mmol/L | 1.7±0.4 | 1.7±0.6 | | 1.7±0.5 | | 0.75 |
| Glucose, mmol/L | 7.0±1.6 | 6.9±2.5 | | 7.0±2.0 | | 0.92 |
| Urea, mmol/L | 22.6±6.9 | 17.2±5.5 | | 20.9±7.0 | | 0.04 |
| Urate, mmol/L | 376.0±56.6 | 350.7±42.3 | | 368.4±53.9 | | 0.26 |
| Creatinine, mmol/L | 1015.0±238.1 | 759.3±205.7 | | 932.5±257.5 | | 0.009 |
| Total protein, g/L | 63.3±6.8 | 62.5±5.6 | | 63.0±6.5 | | 0.74 |
| Albumin, g/L | 34.4±4.3 | 33.5±2.8 | | 34.1±3.9 | | 0.55 |
| Prealbumin, mg/L | 410.4±91.6 | 388.6±117.8 | | 403.9±100.6 | | 0.62 |
| High sensitivity C reactive protein, mg/L | 3.1±2.3 | 21.0±51.0 | | 10.1±33.1 | | 0.94 |
| NT-Pro BNP, pg/ml | 479.3±631.7 | 165.7±107.1 | | 371.2±536.3 | | 0.91 |
| Immunoreactive PTH, pg/ml | 221.8±240.5 | 130.6±81.1 | | 191.4±206.4 | | 0.47 |
| **Dialysate test results** |  |  | |  | |  |
| Kalium, mmol/L | 3.3±0.5 | 3.0±0.5 | | 3.2±0.5 | | 0.13 |
| Natrium, mmol/L | 130.6±3.7 | 128.5±4.9 | | 129.9±4.2 | | 0.22 |
| Calcium, mmol/L | 1.4±0.1 | 1.5±0.1 | | 1.4±0.1 | | 0.27 |
| Phosphorus, mmol/L | 1.0±0.3 | 0.7±0.3 | | 0.9±0.3 | | 0.04 |
| Glucose, mmol/L | 47.6±9.1 | 53.6±9.4 | | 49.6±9.6 | | 0.11 |
| Urea, mmol/L | 19.9±6.2 | 14.5±4.5 | | 18.2±6.3 | | 0.03 |
| Urate, mmol/L | 236.3±36.0 | 199.3±31.3 | | 224.3±38.6 | | 0.01 |
| Creatinine, mmol/L | 646.1±175.1 | 429.4±135.4 | | 576.2±192.2 | | 0.002 |
| Total protein, g/L | 1.7±0.5 | 2.1±0.6 | | 1.8±0.6 | | 0.12 |
| Albumin, g/L | 0.1±0.1 | 0.1±0.1 | | 0.1±0.1 | | 0.5 |
| NT-proBNP, pg/ml | 479.3±631.7 | 165.7±107.1 | | 371.2±536.3 | | 0.91 |
| iPTH, pg/ml | 221.8±240.5 | 130.6±81.1 | | 191.4±206.4 | | 0.47 |
| **M-mode and Two-Dimensional Echocardiography** |  | |  | |  | |
| LVEDD, mm | 50.5±6.1 | 46.1±6.3 | | 49.1±6.5 | | 0.08 |
| LVESD, mm | 33.4±6.9 | 29.9±4.9 | | 32.3±6.5 | | 0.18 |
| LVEF , % | 62.6±10.7 | 61.2±12.7 | | 62.1±11.4 | | 0.76 |
| LVFS, % | 34.5±7.5 | 35.5±3.4 | | 34.9±6.4 | | 0.85 |
| IVST, mm | 13.5±2.3 | 9.5±1.5 | | 12.3±2.8 | | <0.001 |
| IVSE, mm | 7.5±1.9 | 9.5±1.7 | | 7.8±2.0 | | 0.29 |
| LVPWT, mm | 12.7±2.1 | 9.3±1.4 | | 11.6±2.5 | | <0.001 |
| LVWE, mm | 11.8±2.2 | 11.6±1.3 | | 11.8±2.1 | | 0.87 |
| AoSD, mm | 35.8±3.6 | 29.9±1.9 | | 33.8±4.2 | | <0.001 |
| LAAD, mm | 38.6±6.0 | 35.5±4.0 | | 37.6±5.7 | | 0.15 |
| AAoD, mm | 36.6±4.1 | 32.8±3.8 | | 35.5±4.3 | | 0.04 |
| LATD, mm | 44.2±7.1 | 41.8±5.5 | | 43.6±6.8 | | 0.52 |
| LASID, mm | 54.3±7.5 | 53.2±2.7 | | 54.0±6.8 | | 0.76 |
| RATD, mm | 35.0±6.8 | 30.0±5.3 | | 33.9±6.8 | | 0.16 |
| RASID, mm | 47.8±7.9 | 42.7±0.9 | | 467.0±7.5 | | 0.22 |
| RVAD, mm | 24.8±1.8 | 20.3±3.1 | | 22.1±3.4 | | 0.05 |
| RVTD, mm | 33.9±6.0 | 30.0±5.1 | | 33.3±6.0 | | 0.33 |
| MPAD, mm | 25.4±4.0 | 22.2±2.6 | | 24.4±3.9 | | 0.03 |
| **Doppler Echocardiography** |  |  | |  | |  |
| Aortic valve flow-velocity-max, cm/s | 132.5±34.7 | 124.9±19.1 | | 130.1±30.8 | | 0.53 |
| Mitral valve flow-velocity -E peak, cm/s | 82.8±20.9 | 82.1±20.7 | | 82.6±20.8 | | 0.94 |
| Mitral valve flow-velocity -A peak, cm/s | 102.7±21.7 | 79.8±32.1 | | 94.8±28.0 | | 0.04 |
| Pulmonary valve flow-velocity-max, cm/s | 98.6±18.6 | 92.9±18.0 | | 96.8±18.6 | | 0.44 |

Abbreviation: LVEDD, Left ventricular end diastolic diameter; LVESD, Left ventricular end systolic diameter; LVEF, Left ventricular ejection fraction; LVFS, Left ventricular fractional shortening; IVST, Interventricular septal thickness; IVSE, Interventricular septal excursion; LVPWT, Left ventricular posterior wall thickness; LVWE, Left ventricular wall excursion; AoSD, Aortic sinus diameter; LAAD, Left atrial anteroposterior diameter; AAoD, Ascending aorta diameter; LATD, Left atrium transverse diameter; LASID, Left atrial suprainferior diameter; RATD, Right atrial transverse diameter; RASID, Right atrial suprainferior diameter; RVAD, Right ventricular anteroposterior diameter; RVTD, Right ventricular transverse diameter; MPAD, Main pulmonary artery diameter.

**Supplement Table 2. Comparison of body composition between CAPD patients and healthy volunteers.**

| **Characteristic** | **CAPD patients（N=31）** | | **Healthy volunteers（N=310）** | | **P Value** | |
| --- | --- | --- | --- | --- | --- | --- |
|  | **Male (N=21)** | **Female(N=10)** | **Male (N=149)** | **Female(N=161)** | **Male** | **Female** |
| **Age, years** | 56.4±10.6 | 54.7±14.2 | 52.3±12.6 | 47.6±15.3 | 0.16 | 0.16 |
| **Height, cm** | 172.4±3.8 | 155.3±4.5 | 169.9±4.9 | 161.1±4.9 | 0.02 | 0.00 |
| **Weight, kg** | 71.4±7.6 | 53.5±6.3 | 75.7±9.1 | 60.2±10.0 | 0.04 | <0.001 |
| **Protein, Kg** | 11.9±1.4 | 8.5±1.0 | 11.3±1.3 | 8.6±1.1 | 0.04 | 0.63 |
| **Inorganic salts, Kg** | 4.5±0.6 | 3.4±0.4 | 4.0±0.6 | 3.3±0.4 | 0.00 | 0.30 |
| **Body fat, Kg** | 9.0±4.6 | 9.5±3.1 | 18.4±4.8 | 16.0±5.6 | <0.001 | 0.00 |
| **Skeletal Muscle Mass, Kg** | 34.0±4.1 | 23.5±2.9 | 32.1±3.9 | 24.1±3.4 | 0.04 | 0.58 |
| **Body mass index, kg/m^2^** | 24.0±2.6 | 22.2±2.1 | 26.2±2.7 | 23.2±3.3 | 0.13 | 0.37 |
| **Percent Body Fat, %** | 12.7±6.3 | 17.6±4.6 | 21.5±9.3 | 23.9±9.5 | 0.00 | 0.04 |
| **Total body water, TBW, L** | 45.7±5.2 | 32.2±3.7 | 42.1±4.7 | 32.3±4.2 | 0.00 | 0.96 |
| **Intracellular water, ICW, L** | 27.6±3.1 | 19.6±2.2 | 26.1±3.0 | 20.0±2.6 | 0.04 | 0.60 |
| **Extracellular water, ECW, L** | 18.1±2.2 | 12.6±1.6 | 16.0±1.7 | 12.3±1.6 | 0.00 | 0.49 |
| **ECW/TBW, %** | 0.40±0.01 | 0.39±0.02 | 0.38±0.01 | 0.38±0.01 | <0.001 | 0.00 |
| **TBW of Right Arm, L** | 2.2±0.4 | 1.4±0.3 | 2.2±1.1 | 1.9±0.5 | 0.90 | 0.02 |
| **TBW of Left Arm, L** | 2.1±0.4 | 1.4±0.3 | 2.2±1.2 | 1.8±0.5 | 0.88 | 0.01 |
| **TBW of Trunk, L** | 17.6±2.0 | 12.8±1.8 | 17.8±6.3 | 15.7±3.1 | 0.87 | 0.00 |
| **TBW of Right Leg, L** | 9.2±1.3 | 5.6±0.9 | 8.0±2.3 | 7.4±2.3 | 0.02 | 0.02 |
| **TBW of Left Leg, L** | 8.9±1.1 | 5.5±1.0 | 7.8±2.0 | 7.2±2.1 | 0.01 | 0.01 |
| **TBW of Total segment, L** | 40.0±4.3 | 26.7±3.5 | 37.9±12.0 | 33.9±7.9 | 0.44 | 0.00 |

**Supplement Figure 1.** Conventional Bland-Altman plots showing the difference vs. mean value of dry weight assessment on DSM-BIA and Conventional BIA for total healthy patients and both genders. The mean difference and 95% limits of agreement are shown. There are good agreements between the two assessment methods in the measurements of DWA of healthy people, of which 93.5% (8/310) for total people, 96.6% (5/149) for male, 96.3% (5/161) for female, respectively.

**Supplement Table 3. Linear regression analysis of parameters possibly associated with ECW/TCW.**

| **Parameter** | **ANOVA（F）** | **Univariate Liner Regression（Std. β）** | | **P value** |
| --- | --- | --- | --- | --- |
| **Blood test results** |  |  |  | |
| White blood cells, ×10^9^/L | 0.18 | 11.2 | 0.67 | |
| Red blood cells, ×10^12^/L | 0.70 | 6.8 | 0.41 | |
| Hemoglobin, g/L | 2.11 | 478.0 | 0.16 | |
| Kalium, mmol/L | 0.01 | -0.7 | 0.93 | |
| Natrium, mmol/L | 0.02 | -4.8 | 0.88 | |
| Calcium, mmol/L | 1.25 | -3.0 | 0.27 | |
| Phosphorus, mmol/L | 0.00 | 0.3 | 0.96 | |
| Glucose, mmol/L | 3.83 | 47.3 | 0.06 | |
| Urea, mmol/L | 0.01 | 7.7 | 0.93 | |
| Urate, mmol/L | 1.69 | -901.9 | 0.20 | |
| Creatinine, mmol/L | 3.53 | -6040.0 | 0.07 | |
| Total protein, g/L | 2.10 | -119.6 | 0.16 | |
| Albumin, g/L | 3.55 | -91.4 | 0.07 | |
| Prealbumin, mg/L | 3.49 | -2683.0 | 0.07 | |
| High sensitivity C reactive protein, mg/L | 0.04 | -143.5 | 0.84 | |
| iPTH, pg/ml | 0.00 | -125.6 | 0.96 | |
| **Dialysate test results** |  |  |  | |
| Kalium, mmol/L | 0.33 | 3.8 | 0.57 | |
| Natrium, mmol/L | 2.31 | 81.6 | 0.14 | |
| Calcium, mmol/L | 0.32 | 0.7 | 0.57 | |
| Phosphorus, mmol/L | 0.52 | 3.2 | 0.48 | |
| Glucose, mmol/L | 0.61 | -98.0 | 0.44 | |
| Urea, mmol/L | 0.00 | 5.6 | 0.95 | |
| Urate, mmol/L | 0.18 | 214.9 | 0.68 | |
| Creatinine, mmol/L | 0.93 | -2414.0 | 0.34 | |
| Total protein, g/L | 0.68 | -6.0 | 0.42 | |
| Albumin, g/L | 0.00 | -0.04 | 0.98 | |
| **M-mode and Two-Dimensional Echocardiography** |  |  |  | |
| LVEDD, mm | 1.88 | 113.6 | 0.18 | |
| IVST, mm | 0.29 | 19.7 | 0.59 | |
| IVSE, mm | 1.26 | -3973.0 | 0.28 | |
| LVPWT, mm | 0.86 | 29.8 | 0.36 | |
| LVWE, mm | 2.24 | -53.3 | 0.15 | |
| AoSD, mm | 1.78 | 72.6 | 0.19 | |
| AAoD, mm | 2.05 | 81.1 | 0.16 | |
| LATD, mm | 3.65 | 214.8 | 0.07 | |
| LASID, mm | 2.76 | 188.8 | 0.11 | |
| RATD, mm | 1.30 | 113.0 | 0.27 | |
| RASID, mm | 2.19 | 192.7 | 0.16 | |
| RVAD, mm | 0.15 | -31.6 | 0.71 | |
| RVTD, mm | 1.76 | 139.5 | 0.20 | |
| MPAD, mm | 1.12 | 53.3 | 0.30 | |
| **Doppler Echocardiography** |  |  |  | |
| Aortic valve flow-velocity-max, cm/s | 0.06 | 101.1 | 0.81 | |
| Mitral valve flow-velocity -E peak, cm/s | 0.53 | 199.1 | 0.47 | |
| Mitral valve flow-velocity -A peak, cm/s | 2.88 | 616.2 | 0.10 | |
| Pulmonary valve flow-velocity-max, cm/s | 1.58 | 300.8 | 0.22 | |

Abbreviation: LVEDD, Left ventricular end diastolic diameter; IVST, Interventricular septal thickness; IVSE, Interventricular septal excursion; LVPWT, Left ventricular posterior wall thickness; LVWE, Left ventricular wall excursion; AoSD, Aortic sinus diameter; AAoD, Ascending aorta diameter; LATD, Left atrium transverse diameter; LASID, Left atrial suprainferior diameter; RATD, Right atrial transverse diameter; RASID, Right atrial suprainferior diameter; RVAD, Right ventricular anteroposterior diameter; RVTD, Right ventricular transverse diameter; MPAD, Main pulmonary artery diameter.
